# Being an intentional healer: cultural humility approach for African Americans

**DOI:** 10.11604/pamj.2022.41.216.33694

**Published:** 2022-03-16

**Authors:** Ramar Henderson, Monica Lynn Miles, Donna Murray

**Affiliations:** 1 Kremen School of Education and Human Development, California State University, Fresno, California, United States of America,; 2 Physician Assistant Education Association, Washington DC, United States of America

**Keywords:** African Americans, Blacks, cultural competency, healthcare disparities

## Abstract

The purpose of this commentary is to provide health professionals and educators with guiding questions to include into their practice to critically reflect on the care their African American patients receive. African American patients have historically experienced health disparities in comparison to non-African Americans. This dichotomy of care is embedded in racist ideologies and practices. A cultural humility approach is one way to inform the care of African American patients.

## Commentary

The purpose of this commentary is to provide health professionals and educators with guiding questions to include into their practices to critically reflect on the care their African American patients receive. Cultural humility is defined as a process of reflecting (i.e. self), owning, and understanding how an individual´s own culture may impact how they understand what cultural identity is salient to that of the client [1]. Cultural humility is one construct for understanding and developing a process-oriented approach to enhancing your multicultural orientation, regardless of your race, gender, citizenship status, and other identities. Multicultural competence refers to amassing competence, skills, and application that can aid in effective healing [2]. One of the overarching tenets of being a multicultural competent practitioner is that there can be limits on knowledge attainment [3]. Moreover, cultural humility aims to elevate the micro-processes associated with understanding the concept of self and how that impacts the interpersonal connection with the client [4]. In sum, Hook Davis, Owen, and colleagues (2013) conceptualized cultural humility as the “ability to maintain an interpersonal stance that is other-oriented (or open to the other) in relation to aspects of cultural identity that are most important to the (person)” [3].

African Americans as a collective are reluctant to seek medical services from an allied health professional due to a gross amount of distrust in the medical system [5]. For example, historically, the medical profession has had lapses in the collective judgment regarding the services rendered to African Americans [6]. The infamous and egregious, United States Public Health Service Syphilis Study at Tuskegee involved six hundred African American men who were intentionally infected and not treated for syphilis, to satisfy the curiosity of White physicians [7]. The United States government has tried to address unethical medical practices and studies on African Americans, yet the medical field is embedded with racist practices and African Americans are still reluctant to engage with medical systems, due to mistrust and oftentimes improper care [5]. The office of minority health reported in comparison to their White counterparts, African Americans have a shorter life expectancy and have higher rates of heart diseases, stroke, cancer, asthma, influenza and pneumonia, and diabetes [8]. At the same time, the majority of allied health professions have conceptual practices that are rooted in a Eurocentric standard and racist practices. The racist history of medicine is causal to why African Americans have less faith and utilization of services from allied health professionals [6].

A lifelong commitment to self-evaluation and self-critique is essential for understanding racial disparities as a medical and allied health professional. Some conceptual and practical anchors of both the allied and medical professionals are rooted in a Eurocentric model, which can invalidate the lived experiences of African Americans. Researchers have examined the relationship between quality of care and race, extolling the narrative that people of color receive insufficient attention compared to their white American peers. As the current models (e.g. medical, holistic) of care can be embedded with racist practices, so there is a need to critically examine care and perceptions held. Health disparities exist due to the relationship between racism and the inability to examine one´s proximity to racist ideologies (i.e. ideologies that do not center the lived experiences of clients of color). Moreover, the question is often not if health disparities exist but rather what they look like in your current context. In a society where racism is ingrained into our societal structure, the question is not if racism is present but rather what it looks like where you are positioned. Racism is much more than what historical depictions suggest (e.g. Ku Klux Klan burning crosses at African Americans´ homes). Racism is ever-changing and exists in all systems. Both the practitioner and patient must work in collaboration for the result to be positive health outcomes, recognize the practitioner as more institutional and medical knowledge and the patient it the expert on their lives experiences and how they are feeling. African American patients are more likely to have complaints of physical pain go untreated than their white counterparts. This tenant of cultural humility suggests that the evaluation of yourself must include the examination of your proximity to racist ideologies.

Taking a cultural humility approach requires an interdisciplinary understanding of having medical professionals work outside of their discipline with communities to address health disparities. Moreover, practitioners must consider the impact of systems on the overall health of people who live within the margins of humanity. Though individuals can create positive change, communities and groups can also have a profound impact on systems. We cannot individually commit to self-evaluation and fixing power imbalances without advocating within the larger organizations in which we participate. Cultural humility, by definition, is larger than our individual selves we must advocate systemically. For this purpose, we developed the following guiding questions for a cultural humility approach with African Americans: 1) how does my racial identity impact how I view and understand the needs of African Americans in my practice; 2) what do I know about the local African American community both their history and current issues (i.e Flint, Michigan, water crisis, and high lead exposure in patients); 3) how am I promoting active and empathetic listening in African American patients; a) what might it look like through a cross-racial lens (i.e. an inference that other groups will be working with African American clients); 4) how have I critically examined how my profession has reified racist ideologies to maintain health disparities; 5) how am I existing in a profession with Eurocentric conceptual and practical anchors; 6) what groups am I connected to help me stay actively engaged in understanding my profession and the needs of my local African American community members; a) the need for Afrocentric pedagogies (e.g. black psychology).
